# Development of a Vaccine Advocacy Scale for Childhood Vaccines and Psychometric Evaluation: A Methodological Study

**DOI:** 10.1111/jep.70056

**Published:** 2025-03-26

**Authors:** Deniz S. Yorulmaz Demir

**Affiliations:** ^1^ Nursing Department, Faculty of Health Sciences Artvin Çoruh University Artvin Turkey

**Keywords:** advocacy, child advocacy, reliability, vaccine, validity

## Abstract

**Aim:**

This study developed the Vaccine Advocacy Scale for childhood vaccines for adults and evaluated its psychometric properties.

**Method:**

This methodological study involved 211 adults. A literature review was conducted to create the item pool of the scale, and 12 items were prepared. While evaluating the scale's psychometric properties, the researchers performed content validity, explanatory factor analysis (factor loadings of the items, eigenvalues of the sub‐dimensions, and explained variance rates), confirmatory factor analysis (factor loadings and common fit indices), and criterion validity (predictive validity) in the validation phase. In the predictive validity assessment, the distribution of scores on the scale was examined according to some behaviours related to vaccine advocacy. Additionally, we analysed the item‐total score correlation, Cronbach's alpha coefficient, and split‐half test consistency in the reliability phase.

**Results:**

The study's calculated Kaiser‐Meyer‐Olkin value was 0.868, and Bartlett's test of sphericity resulted in significant results (*X* ^2^ = 1724.166; *p* < 0.001). The explanatory factor analysis revealed that the items' factor loadings were between 0.451 and 0.949 and explained 58.29% of the total variance of the structure, which consisted of 12 items and two sub‐dimensions. The confirmatory factor analysis found the factor loadings of the items between 0.62 and 0.85 and identified ‘common fit indices’ within acceptable ranges and close to the perfect fit values (*X* ^2^/df, GFI, CFI, RMSEA, RMR, NFI, TLI and IFI were 1.906, 0.950, 0.952, 0.093, 0.059, 0.906, 940 and 0.953, respectively). The Cronbach's alpha value for the scale was 0.92, and the Spearman‐Brown coefficient, Guttman's split‐half coefficient, and split‐half correlation coefficients were 0.843, 0.842 and 0.713, respectively. The study findings indicated that individuals who had talked to other parents about vaccines, recommended vaccinations, and communicated vaccine‐related issues with medical professionals had significantly higher total scale scores (*p* < 0.005).

**Conclusion:**

Considering the study findings and evaluations, the Vaccine Advocacy Scale was a valid and reliable measurement tool to assess adults’ vaccine advocacy behaviour for childhood vaccines.

## 1 Introduction

Vaccines are one of the most effective public health instruments for reducing infant and child mortality. The World Health Organisation (WHO) estimated that ‘vaccination reduced infant mortality by 45%’. Although vaccines are proven to protect and sustain public health and lower disability and mortality rates, vaccine refusals have been increasing and immunisation rates decreasing globally. WHO reported that 25 million children were unvaccinated or under‐vaccinated in 2021 [1]. As childhood immunisation rates have decreased globally, WHO has included vaccine hesitancy and vaccine refusal among 10 global public health problems that require attention and resolution, recommending that health care professionals receive vaccine education [2]. Although WHO indicates that information provided by health care professionals plays a crucial role in preventing vaccine hesitancy [3], current approaches emphasise that public partnerships with people other than health care professionals advocating for vaccination also play a critical role in preventing vaccine hesitancy [4].

Advocacy refers to ‘supporting the subject by asking more questions to professionals on the subject, seeking more information, and confirming the accuracy of the information obtained, with increasing participation’ [5]. In contrast, vaccine advocacy involves ‘seeking more information about vaccines, encouraging the public to vaccination with scientific information and defending everyone's right to equal access to vaccination’ [4, 5]. Vaccine advocacy is a tool needed globally to ensure equity among members of the public. It is a concept that focuses on reducing infectious diseases and enhancing the quality of life through initiatives that aim to ensure equal access to vaccination for individuals, the implementation of appropriate public health partnerships, and the existence of vaccine advocates [6]. The United Nations recognises vaccines among the Sustainable Development Goals, stating that vaccines are an instrument to ensure healthy living and well‐being for all and that extended vaccination coverage is a goal to help achieve the ideal of a healthy society. The United Nations also emphasises that nations should make the necessary plans to provide and increase vaccine accession rates [7]. Research on vaccine advocacy on vaccine acceptance and public health between 2017 and 2023 has shown that vaccine advocacy by community leaders increases oral polio vaccine uptake [8]. There are also reports indicating parents' decisions to vaccinate their children against HPV have a favourable effect on the vaccination acceptance motivation of other children and parents in schools [9] and that vaccine advocacy for the public is an effective strategy to control the measles epidemic [10]. These results show that vaccine advocacy by community stakeholders such as mothers and religious officials can increase immunisation rates and control infectious diseases and, these results are significant [8, 9, 10].

For vaccination to be successful, it is essential to reach high vaccination rates in society [11]. The increase in vaccine refusals, including in Turkey, has resulted in a decline in immunisation rates. With the decrease in vaccination coverage, infectious disease cases have significantly increased, and outbreaks of vaccine‐preventable diseases, especially measles, have begun to resurface [1, 12]. The contemporary public health perspective holds that responsibility for solving an individual's problem rests with society as a whole. The increase in vaccine refusals, decrease in immunisation rates, and concurrent increase in cases of infectious diseases affect the entire society; as a result, the entire society's responsibility and vaccine advocacy are necessary to address and solve these issues [13, 14].

It is known that negative information about vaccinations spreads among mothers and parents, especially through social media [5, 9]. Having vaccine advocates is crucial in addressing vaccine hesitancy [13, 14]. In light of the available data, community stakeholders can shape vaccine acceptance behaviours, vaccine advocacy appears to be a potent strategy for protecting and sustaining public health, which raises the question of how to assess vaccine advocacy. A literature review on vaccine advocacy–related assessment tools revealed only one measurement tool, which assessed health care professionals' influenza vaccine advocacy behaviour [15]. The availability of a valid and reliable measurement tool to study vaccine advocacy behaviour may help assess society's vaccine advocacy and scrutinise the determining factors of vaccine advocacy. In addition, a measurement tool is needed to evaluate the effectiveness of efforts to develop individuals as vaccine advocates. Thus, a valid and reliable measurement tool for assessing vaccine advocacy behaviour is essential. A measurement tool that evaluates vaccine advocacy can provide significant gains for both researchers and health care professionals. This measurement tool can help assess the role of community stakeholders (e.g., religious officials, mothers and teachers) in immunisation, evaluate changes in behaviour that may occur through support for vaccine advocacy, and, as an important outcome, evaluate changes in vaccination rates. Furthermore, current societal participation‐based approaches to reducing rising vaccine hesitancy and refusals make this requirement more urgent and necessary. Therefore, it is important to have a measurement tool that evaluates the vaccine advocacy behaviour of the community. In addition, despite the increase in scientific studies on vaccine advocacy, there is still a lack of a measurement tool to evaluate vaccine advocacy. Thus, this study developed the following ‘Vaccine Advocacy Scale’ for childhood vaccines for adults as a contribution to the body of literature, which lacks subject‐related research.

## 2 Methods

### 2.1 Research Objective and Type

This methodological study involved designing and developing the Vaccine Advocacy Scale and its psychometric evaluation. Methodological research uses the concepts and structures examined to scale. Methodological research is used to develop measurement tools and ensure their validity and reliability [16]. It also followed the principles of the International Test Commission (ITC) Guidelines for Translating and Adapting Tests when reporting Vaccine Advocacy Scale findings [17].

**Figure 1:**
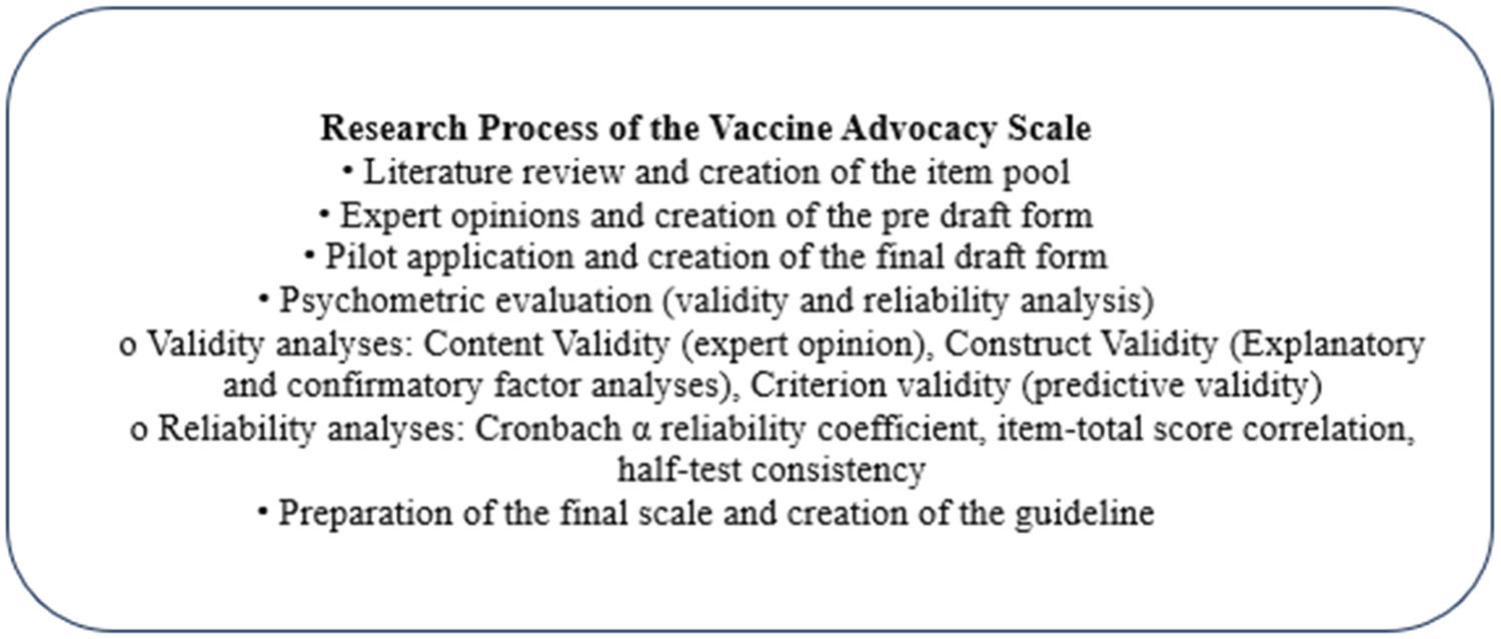
Research process of the Vaccine Advocacy Scale.

The Vaccine Advocacy Scale was developed to evaluate adults' vaccine advocacy behaviour towards childhood vaccines. The research consisted of three stages: (1) the creation of the item pool, (2) the generation of the final draft form with expert opinions and a pilot study, and (3) the psychometric evaluation [16]. Figure 1 displays the steps followed during the research development process for the Vaccine Advocacy Scale.

#### 2.1.1 Stage 1: Literature Review and Item Pool Creation

The researchers performed a literature review using the Google Scholar, PubMed, MEDLINE, Embase, and CINAHL databases to create an item pool for the Vaccine Advocacy Scale using the following keywords separately and in combination: ‘vaccine, advocacy, vaccine advocacy’. The literature review was conducted between October 1 and 30, 2023, and occurred in the English language. During the item pool creation process, the researchers also analysed a scale assessing influenza vaccine advocacy among health care professionals and referring to the definitions of advocacy and vaccine advocacy [5, 6, 15]. Additionally, during the item pool creation, various measurement tools for parents and adults—such as the Public Attitude Towards Vaccination Scale—Health Belief Model, Vaccine Literacy Scale, and Parent Attitudes about Childhood Vaccines were examined [18, 19, 20]. These measurement tools were examined to differentiate the concept of vaccine advocacy from concepts that influence vaccine behaviour processes (e.g., literacy, attitude and hesitancy). During the literature review and item pool creation process, a document from the WHO regarding vaccine advocacy was also examined [21]. As provided below, the literature review resulted in a 12‐item pool to develop the Vaccine Advocacy Scale.

1. I follow current updates on childhood vaccinations.
2. I question the reliability of the data I obtained about childhood vaccines.
3. I read reports on childhood vaccines from official organisations such as the WHO and the Ministry of Health.
4. I communicate with medical professionals about childhood vaccines, including physicians and nurses.
5. I discuss the significance of childhood vaccines with other individuals/parents.
6. I can respond to questions people may ask regarding childhood vaccines.
7. I can recommend reliable information sources to parents about where to get information about childhood vaccines.
8. I encourage parents to have their children vaccinated on time.
9. I inform parents that, in addition to the commonly known childhood vaccines such as measles and chickenpox, vaccines such as rotavirus, meningitis, and HPV are also available.
10. I publicly mention that childhood vaccination is a significant practice.
11. I advocate that all children in a society should have equal access to vaccination.
12. I follow/read scientific data about childhood vaccines.

#### 2.1.2 Stage 2: Generation of the Final Draft Form With Expert Opinions and Pilot Study

After creating the item pool for the scale, researchers received expert opinions to assess the items' clarity, readability, and eligibility to measure the desired conditions. Five experts with doctorates in public health, paediatrics, and psychiatric nursing and research experience in child health, vaccination, immunisation, measurement tool development, and adaptation evaluated the items in the pool. The Davis technique (1: irrelevant, 2: serious editing necessary, 3: very little modification required, 4: appropriate) was used to evaluate the expert opinions [22]. After obtaining the expert opinions, the researchers prepared a pre‐draft form by making minor edits to the items, without removing any from the item pool. Subsequently, they performed a pilot study on 30 adults and received their feedback for evaluation. As a result, the final draft form was created by making minor changes to the items based on the adults' feedback.

#### 2.1.3 Stage 3: Psychometric Evaluation

The psychometric evaluation phase initially involved the analysis of the item‐total correlation values, which were determined to be 0.32, considering the available literature on item selection [23]. Explanatory factor analysis (EFA) and confirmatory factor analysis (CFA) were used to analyse the scales' construct validity. While the EFA stage involved analysis of the factor loadings of the items, eigenvalues of the sub‐dimensions, and explained variance rates, the CFA stage included evaluation of the factor loadings and common fit indices to determine whether the structure of the EFA was compatible with theoretical knowledge. In the predictive validity phase, the distribution of scores on the scale was examined according to some behaviours related to vaccine opinion and advocacy (e.g., communicating with health professionals about vaccines, reading reports of official organisations, evaluating the accuracy of information, wanting to inform other parents about vaccines). The Cronbach's alpha reliability coefficient and two‐half‐test consistency (Spearman‐Brown and Guttman's coefficient) revealed the scale's reliability. Following all the analyses, the researchers developed the final version of the Vaccine Advocacy Scale and the guidelines for its use.

### 2.2 Data Collection Tools

The Descriptive Features Questionnaire and Vaccine Advocacy Scale developed based on the literature review were used to collect the study data [5, 6, 8, 9, 15, 18, 19, 20, 24, 25].

#### 2.2.1 Descriptive Features Questionnaire

This questionnaire consisted of 12 questions assessing the participants' sociodemographic features (age, gender, marital status, having a child/children, individual vaccination practices, vaccination status of the parents, vaccination status of the children), vaccine‐related opinions (confidence in vaccines and considerations regarding whether should be mandatory/optional), and some health behaviours (communicating with health care professionals about vaccines and reading reports of government agencies).

#### 2.2.2 Vaccine Advocacy Scale

This scale aimed to assess the vaccine advocacy behaviours of adults toward childhood vaccines. The responses to the items on the scale were as follows: 1: Never, 2: Rarely, 3: Occasionally, and 4: Mostly. The scale included no reverse‐scored items, and the total score was calculated by summing the response scores of each item. The scale had no cut‐off point, and the total scale score ranged between 12 and 48. A higher score on the scale indicates higher vaccine advocacy behaviour.

### 2.3 Research Population

The literature on scale development and adaptation studies revealed that there should be 5−10 participants for each item in the sample calculation [26]. Based on this, 60−120 participants were required for the psychometric evaluation of the Vaccine Advocacy Scale. Also, the literature on scale development and adaptation studies indicated the need for EFA and CFA analyses on different data sets [26]. Therefore, the study aimed to reach 120−240 participants and included 211 adults in the research process.

### 2.4 Data Collection

The researchers collected the study data between March 1 and May 1, 2024, from two family health centres (FHCs) in the city centre where the research was conducted and where the community frequently seeks primary health care. They approached adults who presented at the FHCs, explained the purpose and scope of the research, and provided data collection forms to those who volunteered. It took participants five to 6 min to complete the data collection forms. Researchers collected the study data after receiving approvals from the ethics committee and the institution.

### 2.5 Inclusion and Exclusion Criteria

The inclusion criteria were, (1) applying to the health institution where the research data were collected between the dates of the research, (2) not having communication problems, and (3) being a volunteer. Individuals who refused vaccinations for themselves or their children and did not participate in the study were excluded.

### 2.6 Data Analysis

The study used the Statistical Package for the Social Sciences (SPSS) version 25 (v.25) and Analysis of Moment Structures (AMOS) version 25 (v.25) package programs to analyse the research data. It assessed the descriptive data using numbers and percentages and calculated the content validity index (CVI) to evaluate expert opinions. The study also used the Kaiser−Meyer−Olkin (KMO) coefficient and Bartlett's test of sphericity to analyse the eligibility of the data set for factor analysis and the adequacy of the sample size. EFA and CFA revealed the construct validity of the Vaccine Advocacy Scale. While EFA was used to interpret the item factor loadings and the explained variances, CFA was used to calculate the common fit indices (CMIN, CMIN/DF, RMSEA, GFI, CFI, NFI, TLI and IFI).

For item selection, the study considered the item factor loading to be 0.32 and 0.45 in EFA and CFA, respectively [23, 27]. During the data analysis phase, researchers divided the data set into two equal parts (the first half and the last half) and used 106 and 105 items of data in the EFA and CFA analyses, respectively. While evaluating the scale's criterion validity, the researchers examined the change in the scale score according to opinions on vaccination and some health behaviours using an independent group *t*‐test and an ANOVA. The Tukey test was also run to analyse the inter‐group differences between groups of three or more in the ANOVA test. The reliability of the Vaccine Advocacy Scale was evaluated based on item‐total score correlation, Cronbach's alpha reliability coefficient, and split‐half test consistency (Spearman‐Brown and Guttman's coefficient). There was no incomplete or missing data in the process; hence, the study used no completion method for missing data. The study did not include the pilot study data in the analysis. Finally, the study allowed for a 0.05 margin of error in interpreting all analysis results.

### 2.7 Ethical Dimension

Researchers received ethics committee approval (number: E–18457941–050.99–113253; date: 10.11.2023) and institutional permission (number: E–17720518–619–235087692; date: 24.01.2024) before conducting the research, informed participants about the study purpose and content during the research, and received participants' written consent. They also adhered to the principles of the Helsinki Declaration throughout the research.

## 3 Results

The study revealed that 57.3% of the participants had a bachelor's degree or higher and that 93.4% of them had children, 56.9% of whom were between 0 and 5 years old. Regarding the participants' opinions about childhood vaccines, the study found that 65.4% had partial confidence in vaccines or had some reservations, 48.8% believed that vaccines were helpful but not always required, and 55.9% thought that vaccination should be optional. Regarding the vaccination‐related health behaviours of the participants, the study discovered that only 32.7% had received vaccination‐related training, 38.4% recommended vaccination to other parents, and 65.4% assessed the accuracy of the information they acquired about vaccinations (Supporting Information S1: Table 1).

Five experts evaluated the content validity of the item pool created for the Vaccine Advocacy Scale. The scores given by experts to all items were 3 and 4, and the CVI value of the items in the item pool was between 0.88 and 1.00. The CVI value of the entire item pool was 0.95 (Supporting Information S1: Table 2).

### 3.1 Item Reliability

The draft scale's item‐total correlations were analysed, and the results showed that the items' correlation values varied from 0.381 to 0.918. As a result, the study eliminated no items from the item pool (Table 1).

**Table 1:** Item‐total correlations.

Item no	Item correlation	Item no	Item correlation	Item no	Item correlation
I‐1	0.671	I‐5	0.497	I‐9	0.647
I‐2	0.486	I‐6	0.449	I‐10	0.918
I‐3	0.611	I‐7	0.381	I‐11	0.535
I‐4	0.559	I‐8	0.733	I‐12	0.508

The study analysed the KMO value and Bartlett's test of sphericity to evaluate the sampling adequacy of the data set and its eligibility for factor analysis. The KMO value was calculated as 0.868, and Bartlett's test of sphericity provided statistically significant results (*X* ^2^ = 1724.166; *p* < 0.001).

**Figure 2:**
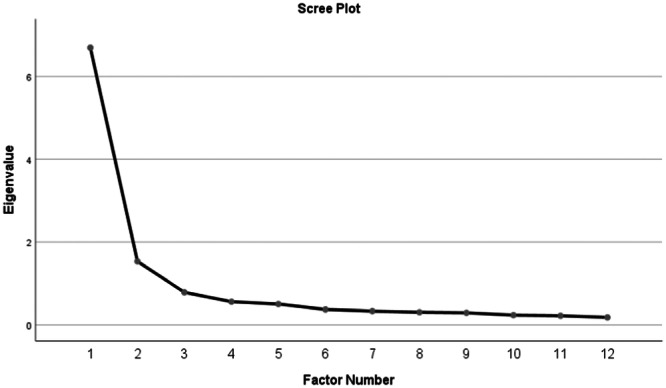
Vaccine Advocacy Scale scree‐plot.

The study performed EFA using the direct oblimin method. This oblique rotation method assumes that the factors are related, to examine the construct validity of the draft scale [23]. The study examined the factor loadings of the items and the eigenvalues of the factors in the EFA stage. The literature review suggested that the factor structure of the measurement tool should potentially be analysed with the maximum likelihood method, which aims to identify the best model with the highest probability for measurement tools [27]. Hence, this study used the maximum likelihood method to assess the scale's factor structure. The study examined the factor loadings of the items and the eigenvalues of the factors in the EFA stage. The factor loading of the items in the draft scale was above 0.32, they displayed no overlapping features, and there were two factors with eigenvalues greater than one. The first factor consisted of eight items, with factor loadings ranging between 0.862 and 0.451. The second factor consisted of four items, with factor loadings varying between 0.949 and 0.710. Consequently, the study found that the first and second factors explained 44.28% and 14.06% of the total variance, respectively. Therefore, the scale explained 58.29% of the total variance (Table 2). Figure 2 displays the scree‐plot graph of the scale.

**Table 2:** Item factor loading values and percentage of variance explained.

Item no	Factor loading	
	Factor 1	Factor 2
I‐1	0.862	
I‐3	0.836	
I‐2	0.730	
I‐12	0.653	
I‐6	0.627	
I‐4	0.553	
I‐7	0.495	
I‐5	0.451	
I‐10		0.949
I‐8		0.864
I‐9		0.781
I‐11		0.710
Total	44.248	14.046
Variance	58.293	

**Figure 3:**
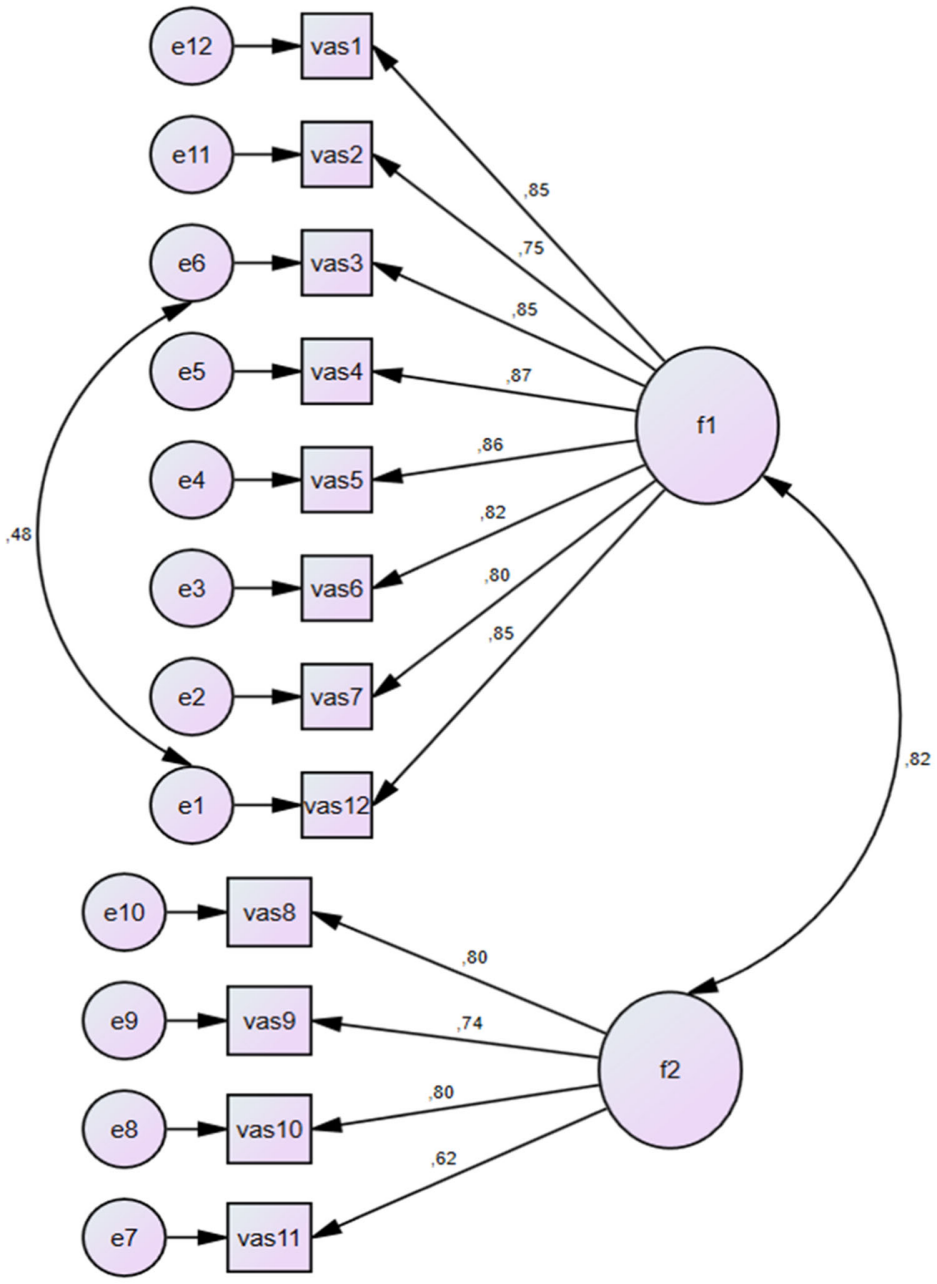
CFA result in and PATH diagram.

Following the analysis of items under the sub‐dimensions, the study named the first and second sub‐dimensions the ‘personal development sub‐dimension’ and the ‘social advocacy sub‐dimension’, respectively, based on the content of the items, available literature on the topic, and expert opinions. The study also performed CFA to evaluate whether the two‐factor structure obtained in the EFA phase was consistent with theoretical knowledge. Table 3 indicates common fit index values and scale values. According to the CFA, *X* ^2^, df and *X* ^2^/df values were 99.096, 52 and 1.906, respectively. The fit indices were 0.950, 0.952, 0.093, 0.059, 0.906, 940 and 0.953 for GFI, CFI, RMSEA, RMR, NFI, TLI and IFI, respectively (Table 3). The CFA factor loadings were between 0.85 and 0.75 for the first sub‐dimension and between 0.80 and 0.62 (Figure 3) for the second sub‐dimension. Additionally, the study used the Hoelter value to evaluate the adequacy of the CFA sample size. The Hoelter values were 74 and 83 individuals at 0.05 and 0.01 significance levels, respectively. Therefore, the researchers concluded that the sample size was sufficient for the CFA.

**Table 3:** Acceptable fit, perfect fit, and scale values for CFA.

Fit index	Acceptable fit	Perfect fit	Scale values
*X* ^2^/df	< 5	< 2	1.906
GFI	> 0.90	> 0.95	0.950
CFI	> 0.90	> 0.95	0.952
RMSEA	< 0.05	< 0.08	0.093
RMR	< 0.05	< 0.08	0.059
NFI	> 0.90	> 0.95	0.906
TLI	> 0.90	> 0.95	0.940
IFI	> 0.90	> 0.95	0.953

Abbreviations: *χ* ^2^, Chi‐square, *χ* ^2^/sd, Chi‐square/degrees of freedom; CFI, comparative fit index; df, degrees of freedom; GFI, goodness of fit index; IFI, Incremental Fıt Indeks (Assessment was conducted in the default model); NFI, normed fit index; RMR, root mean square residual; RMSEA, root mean square error of approximation; TLI, Tucker Lewis Index.

The study used the predictive validity method to evaluate the criterion validity of the Vaccine Advocacy Scale. It also analysed the score distribution of the scale based on some health behaviours related to vaccine opinion and advocacy. These analyses revealed that individuals who opined that childhood vaccines were beneficial and essential practices, had confidence in vaccines, and believed that vaccinations should be mandatory had considerably higher total scale scores (*p* < 0.005). Correspondingly, an examination of the total scale score based on health behaviours also revealed that those who were able to talk about vaccines with different parents, advise vaccination, consult medical professionals about vaccine‐related issues, read government agencies' reports on vaccines, and verify the accuracy of the information they learned about vaccines had significantly higher total scale scores (*p* < 0.005) (Table 4).

**Table 4:** The distribution of Vaccine Advocacy Scale total scores by vaccine opinions and some health behaviours (*n* = 211).

Characteristics	Vaccine Advocacy Scale (total score)		Personal development subscale (f1)		Social advocacy subscale (f2)	
	X ± SD	Test value *p*	X ± SD	Test value *p*	X ± SD	Test value *p*
*Childhood vaccination confidence*						
Yes^1^	36.43 ± 8.32	*F*: 11.256	23.58 ± 5.99	*F*: 6.801	12.84 ± 2.79	*F*: 17.903
Partially^2^	30.18 ± 8.80	< 0.001	19.89 ± 6.14	< 0.001	10.28 ± 3.60	< 0.001
No^3^	28.5 ± 8.23	1 > 2,3	20.54 ± 6.23	1 > 2,3	7.95 ± 3.74	1 > 2.3
*Thoughts about childhood vaccination*						
To be mandatory	34.08 ± 9.12	*t*: 3.777	21.80 ± 6.24	*t*: 1.959	12.27±3.49	*t*: 6.106
To be optional	29.49 ± 8.48	< 0.001	20.11 ± 6.24	0.051	9.38 ± 3.36	< 0.001
*Childhood vaccination idea*						
Useful and necessary^1^	35.41 ± 8.61	*F*: 11.312	22.58 ± 6.18	*F*: 5.532	12.82 ± 3.06	*F*: 24.965
Useful but not all necessary^2^	29.87 ± 8.11	< 0.001 1 > 2,3,4	20.16 ± 5.81	< 0.001 1 > 2,4	9.70 ± 3.27	< 0.001
Unnecessary and harmful^3^	27.00 ± 7.25		21.00 ± 7.08		6.00 ± 2.30	1> 2,3,4
No idea^4^	24.93 ± 10.23		16.37 ± 6.72		8.56 ± 3.79	
*Communicating with health professionals about vaccines*						
Yes	33.17 ± 8.46	*t*: 5.213	21.97 ± 5.82	*t*: 5.027	11.20 ± 3.57	*t*: 4.069
No	25.87 ± 8.75	< 0.001	17.06 ± 6.37	< 0.001	8.81 ± 3.59	< 0.001
*Reading reports of official organisations*						
Yes	35.66 ± 7.21	*t*: 7.000	23.98 ± 4.89	*t*: 7.752	11.68 ± 3.33	*t*: 3.927
No	27.78 ± 8.93	< 0.001	18.04 ± 6.08	< 0.001	9.73 ± 3.79	< 0.001
*Evaluating the accuracy of information*						
Yes	34.07 ± 8.08	*t*: 6.112	22.77 ± 5.60	*t*: 6.699	11.29 ± 3.62	*t*: 3.532
No	26.68 ± 8.83	< 0.001	17.23 ± 5.92	< 0.001	9.45 ± 3.58	0.001
*Talking to another parent about vaccines*						
Yes	35.01 ± 7.57	*t*: 7.278	23.51 ± 5.03	*t*: 8.144	11.50 ± 3.69	3.974
No	26.81 ± 8.75	< 0.001	17.28 ± 6.05	< 0.001	9.52 ± 3.42	< 0.001
*Getting recommendation for childhood vaccines*						
Yes	37.08 ± 7.33	*t*: 8.065	23.90 ± 5.20	*t*: 5.996	13.18 ± 2.84	*t*: 9.258
No	28.04 ± 8.26	< 0.001	18.96 ± 6.17	< 0.001	9.08 ± 3.29	< 0.001
*Want to inform other parents about vaccines*						
Yes	35.32 ± 7.75	*t*: 5.644	23.17 ± 5.48	*t*: 4.867	12.14 ± 3.34	*t*: 5.340
No	28.68 ± 8.92	< 0.001	19.13 ± 6.30	< 0.001	9.55 ± 3.58	< 0.001

Abbreviations: *F*, One Way ANOVA; Mean, Mean, SD, Standard deviation; *t*, Independent samples *t*‐test.

#### 3.1.1 Reliability

The study evaluated the reliability of the Vaccine Advocacy Scale, item‐total score correlation, Cronbach's alpha reliability coefficient (*α*), and split‐half reliability. The item‐total score correlation coefficients were between 0.532 and 0.778 for the entire scale, 0.556 and 0.683 for the personal development sub‐dimension, and 0.499 and 0.707 for the social advocacy sub‐dimension (Table 5). The Cronbach's *α* value was 0.92 (12 items) for the entire Vaccine Advocacy Scale, and it was 0.92 and 0.87 for the individual development and social advocacy sub‐dimensions, respectively. The Cronbach's *α* value was 0.90 and 0.87 for the first and second halves of the scale, respectively. Based on the split‐half reliability test results, the Spearman‐Brown coefficient was 0.843, Guttman's split‐half coefficient was 0.842, and the correlation coefficient between the equivalent halves was 0.713 (Table 6).

**Table 5:** Item‐total and item‐subtotal score correlation.

Item no	Item‐total (*r*)	Item‐subtotal score (*r*)	Item no	Item‐total (*r*)	Item‐subtotal score (*r*)
**I‐1**	0.710	0.649	**I‐7**	0.686	0.556
**I‐2**	0.619	0.562	**I‐8**	0.639	0.654
**I‐3**	0.688	0.683	**I‐9**	0.682	0.599
**I‐4**	0.778	0.682	**I‐10**	0.716	0.707
**I‐5**	0.764	0.636	**I‐11**	0.532	0.499
**I‐6**	0.696	0.594	**I‐12**	0.743	0.672

**Table 6:** Cronbach's alpha and split‐half reliability (*n* = 211).

Scale and subscale	Cronbach's *α*	The first half of Cronbach's *α*	The second half of Cronbach's *α*	Spearman‐brown	Gutman split half	Correlation between two halves
Scale total	0.926	0.903*	0.871**	0.843	0.842	0.713
Personal development	0.922					
Social advocacy	0.877					

* First half: Item 1,2,3,4,5, ve 6.

** Second half: Item 7,8,9,10,11 ve 12.

## 4 Discussion

For many years, vaccines have been the most effective public health measures for controlling infectious diseases and preventing deaths. Yet, vaccine mistrust has recently increased, and vaccine hesitations and refusals are threatening public health [28]. While SAGE acknowledges that educational initiatives and informational campaigns are necessary to prevent vaccine hesitation [3], current strategies focus on establishing public partnerships that support vaccine advocacy [29]. The literature review indicates that it is possible to increase immunisation rates and control infectious diseases through vaccine advocacy [8, 10]. However, there is no measurement tool available to evaluate vaccine advocacy. Structured measurement tools are required to assess vaccine advocacy. Therefore, this study focused on developing the Vaccine Advocacy Scale, a methodological evaluation that rates vaccine advocacy behaviour for childhood vaccines.

Validity is one of the essential features of a measurement tool and refers to the degree to which the tool serves its purpose. It is possible to evaluate validity using different methods, including content validity, construct validity, and criterion validity [30]. Developing the measurement tools, creating an item pool, receiving expert opinions, and ensuring content validity are the critical initial stages of the process. CVI is a value interpreted by evaluating expert opinions during the content validity phase; it indicates the adequacy of the items in the measurement tool for measuring the targeted concept. According to the literature, the CVI value for each item in a measurement tool should have a CVI value of 0.80 or higher, and any item that falls below this threshold should be excluded [22]. This study reviewed the literature to create the item pool of the scale and received opinions from five experts about the items. The study found the CVI values of the individual items to be between 0.88 and 1, with an overall CVI value of 0.95 for all items (Supporting Information S1: Table 2). Thus, the study verified the scale's content validity, concluding that it could adequately measure the targeted concept.

Before evaluating the scale's construct validity, the study analysed the item‐total score correlation values expressing item reliability, the eligibility of the data set for factor analysis, and the adequacy of the sample size. Item‐total score correlation explains the relationship between the score obtained from each item and the total test score. According to the literature, the items of a measurement tool should have item‐total score correlation values greater than 0.32 [31]. A high item‐total score correlation coefficient indicates that the items are homogeneous and the measurement tool adequately evaluates the targeted concept [32]. Regarding the item‐total score correlations of the draft scale, the correlation coefficient of each item in the scale was higher than 0.32. The study also evaluated the eligibility of the data set for factor analysis and the adequacy of the sample size using the KMO value and Bartlett's test of sphericity. According to the literature, the KMO value should be greater than 0.60, and Bartlett's test of sphericity should be significant [33]. This study found the KMO value to be 0.868, and Bartlett's test of sphericity was statistically significant (*X* ^2^ = 1724.166; *p* < 0.001). In line with these findings, the study concluded that the items could successfully evaluate the targeted concept, the data set was appropriate for factor analysis, and the sample size was adequate.

Construct validity, evaluated by factor analyses (EFA and CFA), is a strong indicator of the extent to which a measurement tool can measure the theoretically targeted structure [34]. EFA evaluates the factor loadings of the items, the sub‐dimensions of the measurement tool, and the explained variance rate. In the EFA, items' factor loadings should be at least 0.32 [23] and should explain at least 50% of the total variance in multidimensional scales [35]. In the CFA, however, the compatibility of the structure obtained in the EFA with theoretical knowledge is interpreted [23]. The study initially analysed the construct validity of the Vaccine Advocacy Scale using EFA and subsequently evaluated its compatibility with the theoretical knowledge obtained by CFA. Accordingly, the EFA results for the Vaccine Advocacy Scale revealed that the factor loadings of the items were higher than 0.32, the items were clustered under two sub‐dimensions with eigenvalues greater than 1.00, and the resulting structure explained approximately 60% of the total variance. Thus, the researchers named the first and second sub‐dimensions, ‘personal development sub‐dimension’ and ‘social advocacy sub‐dimension’, respectively, based on the content of the items under the sub‐dimensions and considering the findings in the literature and expert opinions. The concept of advocacy refers to ‘advocating the subject by asking more questions to the professionals of the subject, seeking more information, and confirming the accuracy of the information obtained, with increasing participation’ [5] whereas the concept of vaccine advocacy is ‘encouraging the public to vaccination with scientific information and defending everyone's right to equal access to the vaccination’ [4]. Considering the definitions of these concepts, it is viable to interpretively conclude that the measurement tool retained sufficient content to evaluate the targeted structure. Furthermore, the high rate of the explained variance also supports this interpretation.

CFA is an alternative approach that evaluates a measurement tool's construct validity using typical goodness‐of‐fit indices, such as *χ* ^2^/df, GFI, AGFI, RMSEA, RMR, SRMR, NFI, IFI and TLI. The literature reportedly interprets these fit indices as follows: *χ* ^2^/df ≤ 5 and *χ* ^2^/df ≤ 2 are acceptable and good fits, respectively; GFI, CFI, NFI, TLI and IFI > 0.90 and > 0.95 indicate acceptable and good fits, respectively; and the RMSEA and RMR < 0.05 and < 0.08 are acceptable and good fits, respectively [26]. The literature also recommends item factor loadings of 0.45 and above while selecting items in the CFA [27]. In line with the literature results, this study identified excellent values for *χ* ^2^/df, GFI, CFI, RMSEA, and IFI fit indices and good or almost excellent values for RMR, NFI and TLI fit indices (Table 3). The study also revealed that the factor loadings of the items in the sub‐dimensions were above 0.45 and identified a strong relationship between the two factors. Within the parameters of the CFA results, it is possible to conclude that the structure obtained in the EFA phase is consistent with the literature and capable of measuring the theoretical structure. All these findings support the construct validity of the Vaccine Advocacy Scale and indicate that the developed scale can successfully evaluate adults' vaccine advocacy behaviour for childhood vaccines.

Criterion validity is another strategy for assessing the reliability of measurement tools. There are two different methods for this validity technique: concurrent validity and predictive validity. Predictive validity evaluates the relationship between the measurement result and the potential scenario; in other words, it analyzes the reflection of the measurement result in real life. Predictive validity is a significant technique that provides insight into the requirements for validating a measuring tool in real life [36]. Because the concepts of advocacy and vaccine advocacy involve both individual gain and publicly advocation for the benefit and relevance of vaccination practice for social gain, this study explored the relationship between the vaccine consideration‐related total score of the Vaccine Advocacy Scale and certain health behaviours. Accordingly, the analyses revealed that both the total scale score and the total score for each sub‐dimension were significantly higher among individuals who displayed confidence in vaccines, believed that vaccination should be compulsory, and opined that childhood vaccines were beneficial and should be required. Regarding the total scale score based on health behaviours, the study found that the scale scores of the participants who communicated vaccine‐related issues with medical professionals, read official organisations' reports, and evaluated the accuracy of information was significantly higher than those of other participants. Additionally, the participants who discussed childhood vaccines with other parents, advised parents to make sure their children were vaccinated, and aspired to inform other parents about childhood vaccines had statistically and significantly higher total scale scores and total scores for the sub‐dimensions (Table 4). The subject‐related literature review revealed that vaccination desire is higher in cultures that are aware of the social benefits of vaccines [4], that the strongest predictor of vaccine advocacy is positive attitudes toward vaccines [37], and that the desire for vaccine advocacy is higher among individuals with high positive attitudes toward vaccines and low distrust of vaccine benefits [38]. Therefore, it is possible to infer that the Vaccine Advocacy Scale successfully reflects the real‐life context, and the criterion validity of the scale has been ensured.

Reliability is another essential quality of a measurement tool and indicates the degree of stability, freedom from errors, and consistency of the measurement data. Numerous methods, including Cronbach's alpha coefficient, two‐half test consistency, item‐total score correlation, and test‐retest reliability, are used to assess the reliability of measurement tools [39]. The item‐total score correlation value expresses the relationship between the score obtained from the scale items and the total scale score, providing an acceptable correlation in item selection. The recommended coefficient values in the literature ranged between 0.32 and 0.90 [40]. Analysis of the item‐total score correlation values of the Vaccine Advocacy Scale revealed that the correlation coefficients ranged between 0.532 and 0.778, whereas the correlation values of the items in the personal development and social advocacy sub‐dimensions were 0.556−0.683 and 0.499−0.683, respectively, which were within the range recommended by the literature.

The Cronbach's alpha coefficient is another method used to assess the reliability of measurement tools. Research in the literature reports that the reliability coefficient of measurement tools should be at least 0.70 and that the reliability becomes higher as Cronbach's alpha coefficient gets closer to 1.00 [41]. This study determined that the Cronbach's alpha coefficients for the social advocacy sub‐dimension, the personal development sub‐dimension, and the total Vaccine Advocacy Scale were 0.87, 0.92 and 0.92, respectively. Another method used for reliability analysis in the literature is split‐half test consistency. In this method, the correlation coefficient (*r*) between the two halves of the measurement tool is analysed, and Spearman‐Brown, Guttman's split‐half, and correlation coefficient values between the two halves are interpreted [42]. The literature also reports that if the correlation coefficient is 0.00 ≤ *r* ≤ 0.19, 0.20 ≤ *r* ≤ 0.39, 0.40 ≤ *r* ≤ 0.69, 0.70 ≤ *r* ≤ 0.89, and 0.90 ≤ *r* ≤ 1.00, it indicates no relationship, a poor/low relationship, a moderate relationship, a high relationship, and a very high relationship, respectively, stating that the correlation coefficient should be 0.70 and above in a reliable measurement tool [43]. In the current study, the correlation coefficient between the two halves, the Spearman‐Brown coefficient, and the Guttman's split‐half were all greater than 0.70, according to an analysis of the data. In line with these findings, it is reasonable to infer that the Vaccine Advocacy Scale has a high degree of reliability and evaluates the vaccine advocacy behaviour of adults consistently and accurately.

### 4.1 Limitations

The Vaccine Advocacy Scale has certain limitations, although it is the first valid and reliable measurement tool in the literature that evaluates vaccine advocacy behaviour for childhood vaccines. First, the results could not be compared with those of similar measurement tools because there is no equivalent tool for assessing vaccine advocacy behaviour. Another limitation is that the study sample widely included parents with high education levels. In addition, the validity and reliability of the scale were evaluated using a limited sample size. As a result, this study recommends that future research address this limitation and evaluate the scale's reliability on a less‐educated general population.

## 5 Conclusion

The psychometric evaluation results of the Vaccine Advocacy Scale revealed that it is a valid and reliable measurement tool that can evaluate adults' vaccine advocacy behaviour for childhood vaccines. The scale is potentially applicable to all parents and adults. The Vaccine Advocacy Scale will assist health researchers in evaluating vaccine advocacy behaviour, analysing factors related to vaccine advocacy behaviour, and assessing the impact of vaccination‐based education programs and various vaccine‐related initiatives on vaccine advocacy behaviour. Moreover, psychometric evaluation of the scale in different cultures will significantly contribute to the body of literature and enable international comparisons.

## Author Contributions

The design of the study, literature review, data collection and analysis, writing of the article, supervision, and consultancy were carried out by Deniz S. Yorulmaz Demi̇r. Deniz S. Yorulmaz Demi̇r read and approved the final manuscript.

## Ethics Statement

Artvin Çoruh University (Number: E–18457941–050.99–113253; Date: 10.11.2023). Artvin Provincial Health Directorate (Number: E–17720518–619–235087692; Date: 24.01.2024).

## Consent

During the data collection process, the participants were informed about the research topic and content, and their written consent was obtained. This study was conducted by the principles of the Helsinki Declaration.

## Conflicts of Interest

The author declares no conflicts of interest.

## Supporting information

Supporting information.

## Data Availability

Research data are available upon reasonable request from the corresponding author.
