# Supplementary material for: Development of a Vaccine Advocacy Scale for Childhood Vaccines and Psychometric Evaluation: A Methodological Study
Source: J Eval Clin Pract. 2025 Mar 26;31(2):e70056. doi: 10.1111/jep.70056 (PMC11937728; doi:10.1111/jep.70056)
Supplement: Supplementary file 1 — Supporting information. [file JEP-31-0-s001.docx]

**Supplemental tables**

**Supplemental table 1. Sociodemographic characteristics, vaccination practices and some health behaviors of the participants (n = 211)**

| **Characteristics** | **n** | **%** | **Characteristics** | **n** | **%** |
| --- | --- | --- | --- | --- | --- |
| **Gender**  Female  Male | 152  59 | 72.0  28.0 | **Working status**  Yes  No | 142  69 | 67.3  32.7 |
| **Education (degree)**  Primary and middle school  High school  Bachelor’s | 27  63  121 | 12.8  29.9  57.3 | **Gelir algısı**  Negative income (income < expense)   Neutral income (income = expense)  Positive income (income > expense) | 54  121  36 | 25.6  57.3  17.1 |
| **Having a child**  Yes  No | 197  14 | 93.4  6.6 | **Having a child aged 0-5 (n=197)**  Yes  No | 120  77 | 56.9  36.5 |
| **Vaccination card availability (n=197)**  Available  Not available | 177  20 | 83.9  9.5 | **Childhood vaccinations(n=197)**  Regular and complete  Missing | 45  152 | 22.8  77.2 |
| **Getting a special shot (n=197)**  Yes  No | 60  137 | 30.5  69.5 | **Getting a tetanus shot**  Yes  No | 165  46 | 78.2  21.8 |
| **Getting a Covid-19 shot**  Yes  No | 158  53 | 74.9  25.1 | **Getting a Hepatitis B shot**  Yes  No | 68  143 | 32.2  67.8 |
| **Getting a Hepatitis A shot**  Yes  No | 59  151 | 28.0  71.6 | **Getting an Influenza shot**  Yes  No | 71  139 | 33.6  65.9 |
| **Childhood vaccination confidence**  Yes,  Partially  No | 51  138  22 | 24.2  65.4  10.4 | **Childhood vaccination idea**  Useful and necessary  Useful but not all necessary  Unnecessary and harmful  No idea | 82  103  10  16 | 38.9  48.8  4.7  7.6 |
| **Thoughts about childhood vaccination**  To be mandatory  To be optional | 93  118 | 44.1  55.9 | **Receiving training on vaccines**  Yes  No | 69  142 | 32.7  67.3 |
| **Want to receiving vaccination training**  Yes  No | 102  109 | 48.3  51.7 | **Reading reports of official organizations**  Yes  No | 100  111 | 47.4  52.6 |
| **Receiving information from healtcare professionals about vaccines**  Yes  No | 163  48 | 77.3  22.7 | **Evaluating the accuracy of information**  Yes  No | 138  73 | 65.4  34.6 |
| **Talking to another parent about vaccines**  Yes  No | 121  90 | 57.3  42.7 | **Want to inform other parents about vaccines**  Yes  No | 90  121 | 42.7  57.3 |
| **Getting recommendation for chilhood vaccines**  Yes  No | 81  130 | 38.4  61.6 | **Knowing the herd immunity**  Yes  No | 98  113 | 46.4  53.6 |

n: number, %: percentage.

**Supplemental table 2. Item Scores by Experts (n=5)**

| Item No | X±SD | Min-Max | Content Validity Index |
| --- | --- | --- | --- |
| 123456789101112 | 4.00±04.00±04.00±04.00±04.00±03.80±0.453.80±0.453.80±0.453.80±0.453.80±0.454.00±04.00±0 | 4.00-4.004.00-4.004.00-4.004.00-4.004.00-4.003.00-4.003.00-4.003.00-4.003.00-4.003.00-4.004.00-4.004.00-4.00 | 1.01.01.01.01.00.880.880.880.880.8811 |

X: Mean, SD: Standard Deviation, Min: Minimum, Max: Maksimum.
